# Reporting quality evaluation of the stroke clinical practice guidelines: a systematic review

**DOI:** 10.1186/s13643-021-01805-3

**Published:** 2021-09-30

**Authors:** Shuya Lu, Xufei Luo, Xiaojia Ni, Haoxuan Li, Miaomiao Meng, Yefeng Cai, Yunlan Liu, Mengjuan Ren, Yanrui Sun, Yaolong Chen

**Affiliations:** 1 School of Public Health, Lanzhou University, Lanzhou, 730000 People’s Republic of China; 2 Department of Pediatric, Sichuan Provincial People’s Hospital, University of Electronic Science and Technology of China, Chengdu, 611731 People’s Republic of China; 3 Guangdong Provincial Hospital of Chinese Medicine, The Second Clinical School of Chinese Medicine, Guangzhou, 510120 People’s Republic of China; 4 Guangdong Provincial Academy of Chinese Medical Sciences, Guangzhou, 510120 People’s Republic of China; 5 The Second Clinical Medical College of Lanzhou University, Lanzhou, 730000 People’s Republic of China; 6 Institute of Health Data Science, Lanzhou University, Lanzhou, 730000 People’s Republic of China; 7 Evidence-based Medicine Center, School of Basic Medical Sciences, Lanzhou University, Lanzhou, 730000 People’s Republic of China; 8 Lanzhou University, an Affiliate of the Cochrane China Network, Lanzhou, 730000 People’s Republic of China; 9 Key Laboratory of Evidence Based Medicine and Knowledge Translation of Gansu Province, Lanzhou University, 730000 Lanzhou, People’s Republic of China

**Keywords:** Stroke, Clinical practice guidelines, RIGHT, Report quality

## Abstract

**Objectives:**

To analyze the effectiveness and quality of stroke clinical practice guidelines (CPGs) published in recent years in order to guide future guideline developers to develop better guidelines.

**Participants:**

No patient involved

**Method:**

PubMed, China Biology Medicine (CBM), Wanfang, CNKI, and CPG-relevant websites were searched from January 2015 to December 2019 by two researchers independently. The RIGHT (Reporting Items for Practice Guidelines in Healthcare) checklist was used to assess the reporting quality in terms of domains and items. Then, a subgroup analysis of the results was performed.

**Primary and secondary outcome measures:**

RIGHT checklist reporting rate

**Results:**

A total of 66 CPGs were included. Twice as many CPGs were published internationally as were published in China. More than half were updated. Most CPGs are published in journals, developed by societies or associations, and were evidence-based grading. The average reporting rate for all included CPGs was 47.6%. Basic information got the highest (71.7% ± 19.7%) reporting rate, while review and quality assurance got the lowest (22.0% ± 24.6%). Then, a cluster analysis between countries, publishing channels, and institutions was performed. There were no statistically significant differences in the reporting quality on the CPGs between publishing countries (China vs. international), publishing channels (journals vs. websites), and institutions (associations vs. non-associations).

**Conclusions:**

Current stroke CPGs reports are of low quality. We recommend that guideline developers improve the quality of reporting of key information and improve the management of conflicts of interest. We recommend that guideline developers consider the RIGHT checklist as an important tool for guideline development.

**Trial registration:**

10.17605/OSF.IO/PBWUX.

## Strengths and limitations of this study

### Strengths

Provided a comprehensive systematic review of the reporting quality of stroke CPGs published in journals firstly.

### Limitations

The RIGHT checklist is primarily used for the writing of normative CPGs, which determines the completeness and transparency of reporting in CPGs. It is neither to be used as a measure of quality and internal validity nor as a tool for assessing the quality of reporting in published CPGs.

We searched both the English and Chinese libraries, so that the Chinese guidelines are more complete and account for a higher percentage of the total.

## Background

Stroke, including ischemic and hemorrhagic stroke, is a common and serious global health care problem, the second leading cause of death worldwide [1], and one of the leading causes of death and disability in China [2]. In 1978, the World Health Organization (WHO) defined stroke as a clinical syndrome typically characterized by a rapidly developing focal or systemic disturbance of brain function resulting from a sudden rupture or obstruction of a blood vessel in the brain, lasting more than 24 h or resulting in death [3]. The Global Burden of Disease (GBD) 2016 Stroke Lifetime Risk Collaborators published the results of the global lifetime risk of stroke in *The New England Journal of Medicine*, we found China had the highest estimated risk (39.3%; 95% uncertainty interval, 37.5 to 41.1) among 195 countries and states [4], with more than two million new cases per year according to the China Socio-Demographic Index (SDI) as measured in an article from *The Lancet* [5, 6]. Moreover, the cost of treating stroke places a heavy mental and economic burden on countries and families [7, 8], which is expected to increase further due to an aging population; persistent risk factors, such as high blood pressure; and poor management. Greater than half of China’s population lives in rural areas where the overall incidence of stroke is higher (298 per 100,000 person-years) compared with urban areas (204 per 100,000 person-years) [9]. A study published in *The Lancet* shows that stroke prevalence in rural China increased sharply between 2003 and 2013 [5]. High-quality guidelines can better guide clinical practice. And, standardizing the reporting format of guidelines can facilitate readers’ understanding of guidelines and promote the promotion of guidelines, which are necessary qualities of high-quality guidelines. However, primary medical institutions lack clinical practice guidelines (CPGs) to provide scientific and effective services to stroke patients. Therefore, there is an urgent need to standardize the reporting quality of CPGs for stroke.

With the rapid development of the field of stroke medicine and methodology, the institutions that develop guidelines also paid more attention to stroke. In the past 5 years, many CPGs on stroke have been published in China and internationally. The reporting quality of CPGs greatly affects the reliability of the recommendations of the report and determines the length and breadth of the promotion and dissemination of the guideline, so the reporting quality of the guidelines cannot be ignored. However, relevant research worldwide on the reporting standards and quality evaluation of stroke CPGs is lacking. RIGHT (Reporting Items for Practice Guidelines in Healthcare) checklist is the global reporting standard applicable to guide health policies and systems, public health, and clinical medicine [10]. We intended to use the RIGHT standard to evaluate the reporting quality of stroke CPGs in China and abroad in the past 5 years to provide references for improving the quality and reporting standards formulated by the guidelines.

## Method

### Search strategy

Two researchers searched the following databases independently: PubMed, CBM, Wanfang, China National Knowledge Infrastructure (CNKI), and guideline-related websites, including the National Institute for Health and Care Excellence (NICE), Guidelines International Network (GIN), Scottish Intercollegiate Guidelines Network (SIGN) (http://www.sign.ac.uk), United States Preventive Service Task Force (USPSTF) (https://www.uspreventiveservicestaskforce.org/uspstf/), Canadian Task Force in Preventive Health Care (CTFPHC) (https://canadiantaskforce.ca/), and WHO (http://www.who.int/publications/guidelines/en/). The language restrictions were Chinese or English, and the retrieval time was from January 2015 to December 2019. Before publication, we searched Google Scholar and the official websites of stroke-related journals or organizations to obtain supplemental references, including the WSO (https://www.world-stroke.org/), Stroke Foundation (https://strokefoundation.org.au/), Canadian Stroke Best Practices (https://www.strokebestpractices.ca/), and HSF Canadian Partnership for Stroke Recovery (http://www.canadianstroke.ca/). Additional stroke CPGs that were not included were reviewed until December 2019. A search strategy used the keywords “cerebrovascular*,” “stroke,” “brain*,” “guideline*,” “guidance,” and “guide.” We contacted the official or corresponding author by email to obtain the full text of CPGs that were not available.

### Inclusion and exclusion criteria

#### Inclusion criteria

(1) Related to the theme of stroke; (2) published as a guideline; (3) published in English or Chinese; (4) published in a journal or website.

#### Exclusion criteria

(1) Translation, excerpt, and interpretation of guideline; (2) full text of the guideline is not available; (3) old version of the guideline; (4) bibliographic guideline and guideline proposal; (5) guidelines that are not related to stroke or only contain some stroke content.

### RIGHT statement

The RIGHT checklist is an international standard for guiding the writing and reporting of CPGs for makers of clinical, public health, and other health care fields. RIGHT assists journal editors and peer reviewers in reviewing the CPGs as well as researchers in evaluating and researching them. In contrast to the AGREE checklist, which is dedicated to evaluating the methodological quality of the guideline, the RIGHT checklist is used to assess the reporting quality of the guideline. Therefore, we refer to the quality of the CPGs in the study as the quality of reporting, not the overall quality of the CPGs or methodological quality. In January 2017, the full text of the RIGHT statement was published in the Annals of Internal Medicine and includes 22 items: Basic information (items 1-4), background (items 5-9), evidence (items 10-12), recommendations (items 13-15), review and quality assurance (items 16-17), funding and declaration and management of interests (items 18-19), and other information (items 20-22).

### Literature screening

Six researchers were divided into three groups to extract data separately, and the groups were combined and agreed upon after back-to-back extraction, and negotiated with a third party in case of disagreement. The author explained the screening rules to the researcher before screening the literature, excluded literature that was published too early, read the title and abstract, excluded irrelevant literature, and reserved and included the literature. Furthermore, researchers read the full text of the literature that could be accurately judged. If the eligibility of the article was still not determined, it was submitted to the author and subjected unified processing to determine whether it should be included.

### Data extraction and reporting quality assessment

Before data extraction and formal evaluation, 6 researchers (Shuya Lu, Xufei Luo, Mengjuan Ren, Yunlan Liu, Yanrui Sun, and Ting Zhang) were systematically trained on the RIGHT checklist, and two rounds of pretests were completed to ensure that the researchers’ understanding of each item was consistent. The data extraction table was designed based on the RIGHT checklist. The six fellows were divided into three groups of two each. Every two researchers independently extracted and cross-checked the information of the included CPGs and negotiated with the third party to solve the differences. The extracted data mainly include the following:

(1) The general characteristics of the guideline include title, publication year, country, organization, and whether referred to AGREE and RIGHT checklist.

(2) Each item of the reporting content of the guideline was evaluated as reported,” “partial reported,” “unreported,” and “not applicable.” We evaluated the reporting quality of the included CPGs based on 7 domains and 35 items according to the RIGHT checklist. “Reported”: relevant information is fully presented; “Unreported”: applicable to situations where there is no complete lack of relevant information; “Partially reported”: only part of the content is presented; “Not applicable”: the guideline does not meet the evaluation requirements of an item and cannot be evaluated as “Reported,” “Partially reported,” or “Unreported.”

### Statistical analysis

We used the Microsoft Excel 2016 software and R studio to summarize the reporting rates and percentages of the RIGHT items and domains for the guideline and presented the results graphically. Then, the relevant basic information (source of CPGs, year of publication, whether the CPGs were developed by associations/societies) was analyzed by meta-analysis based on subgroups, and statistical data were processed by the RStudio software. We followed the PRISMA checklist for reporting results [11]. For information that could not be quantified, we performed a narrative analysis.

### Patient and public involvement

No patient involved.

## Results

**Fig. 1:**
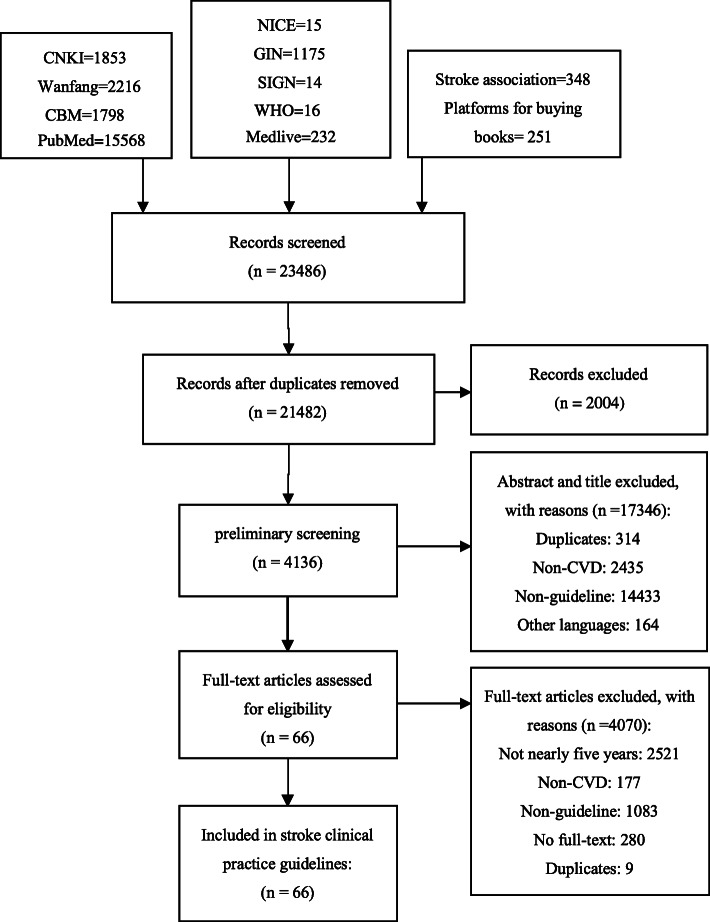
Flow diagram of the selection process for the studies

According to the pre-developed retrieval strategy, a total of 23,486 related literature was obtained, and 66 studies related to stroke were obtained by searching the Chinese and English databases. The literature screening process and results are shown in Fig. 1.

### Basic characteristics of the included CPGs

**Fig. 2:**
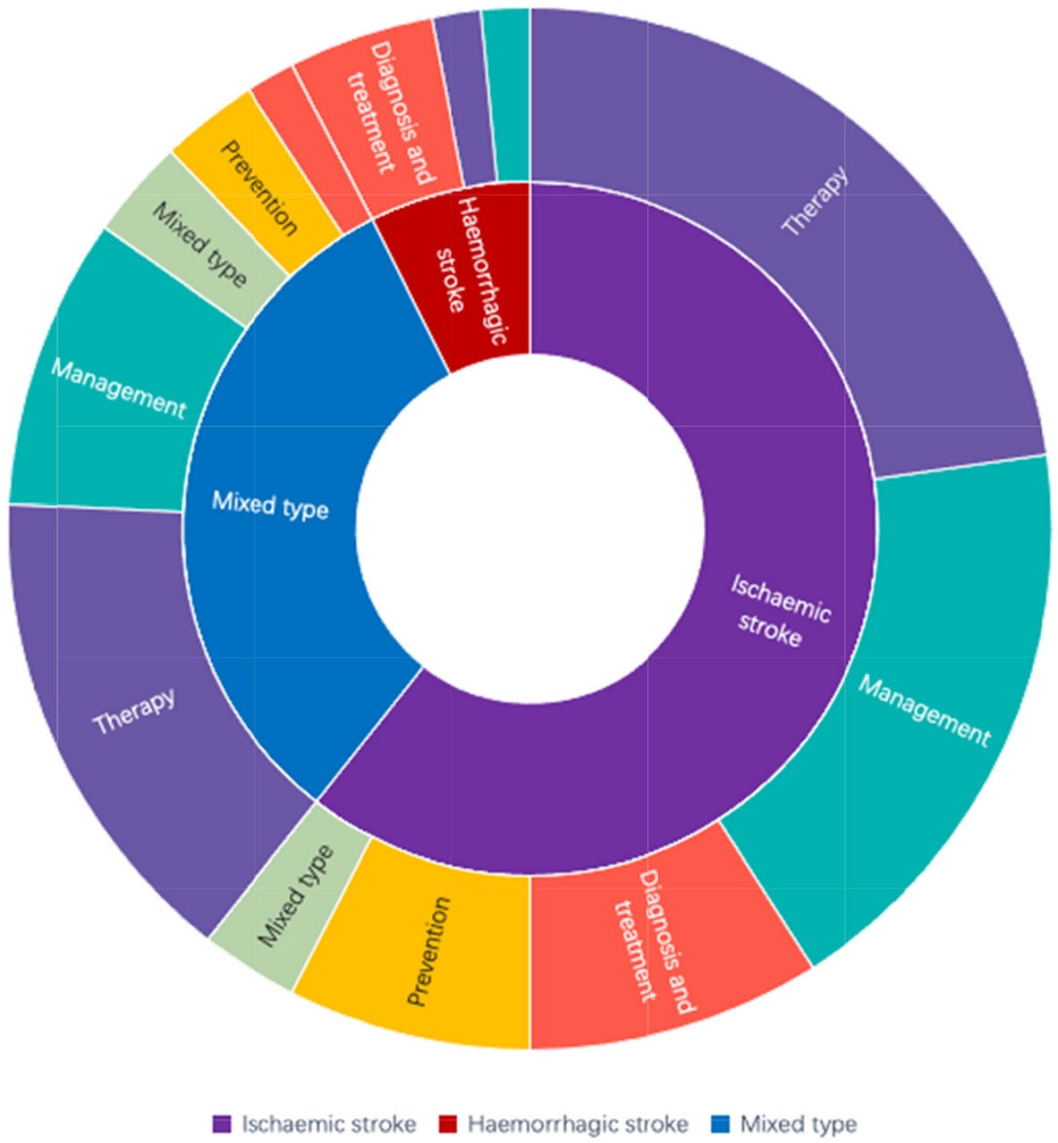
Types of stroke and areas of concern in stroke CPGs

The types of stroke and the distribution of areas of interest in the stroke CPGs are shown in Fig. 2. According to the classification of problems concerned with stroke, we divided the CPGs into five categories with maximum numbers focusing on therapy (39.4%) and management (28.8%) and least on prevention (10.6%). Most of them were ischemic stroke CPGs (60.6%). The basic features of the included CPGs are shown in Table 1. During the past 5 years, China published approximately half as many CPGs (33.3%) as those published abroad (66.7%). Other countries and regions that have published stroke CPGs include the USA (19.7%), Canada (15.2%), Europe (10.6%), Australia (6.1%), Korea (6.1%), the UK (4.6%), Brazil (1.5%), Japan (1.5%), and India (1.5%). Most CPGs were published in 2019, whereas the least were published in 2017 and 2018. Only 15 (22.7%) CPGs were published on the website of the association or society. In terms of developed institutions, most CPGs were produced by various societies or associations (92.4%). According to the type of version, the number of original CPGs (43.9%) was approximately equal to updated CPGs (56.1%). A considerable number of CPGs (83.3%) had an evidence-based rating.

**Table 1:** Characteristics of the CPGs included (*n* = 66) [12–77]

Categories	Number	%
*Country*		
China	22	33.3%
International	44	66.7%
*Publication year*		
2015	14	21.2%
2016	14	21.2%
2017	10	15.2%
2018	10	15.2%
2019	18	27.3%
*Publication*		
Journal	51	77.3%
Website	15	22.7%
*Organization*		
Societies/associations	61	92.4%
Non-societies/associations	5	7.6%
*Type of version*		
Original	29	43.9%
Updated	37	56.1%
*Evidence-based grading*		
Yes	55	83.3%
No	11	16.7%

### Quality of reporting

**Fig. 3:**
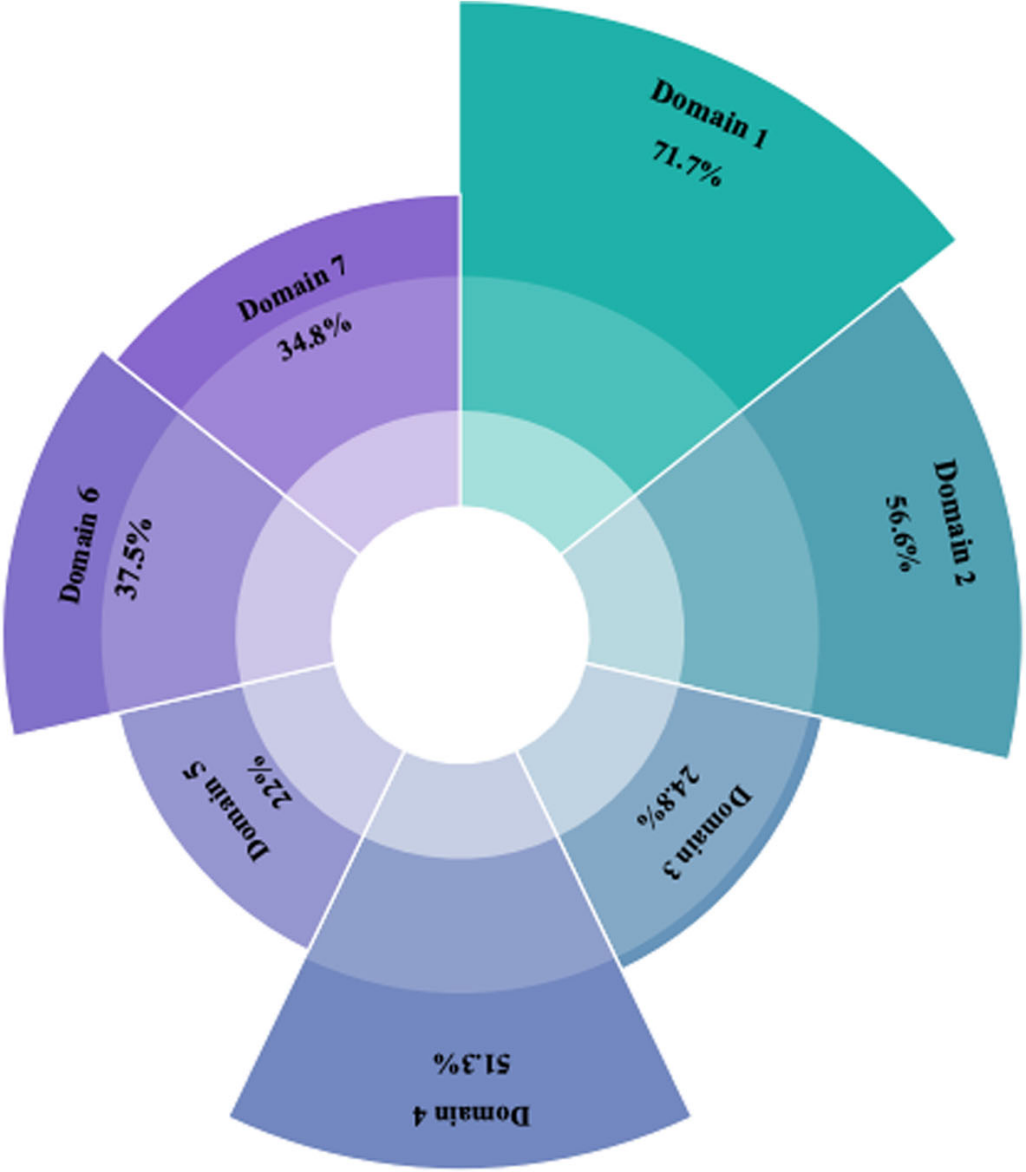
Distribution of reporting rates by domain in the Clinical Guidelines RIGHT checklist. Domain 1: basic information; domain 2: background; domain 3: evidence; domain 4: recommendations; domain 5: review and quality assurance; domain 6: funding, declaration, and management of interest; domain 7: other information

**Fig. 4:**
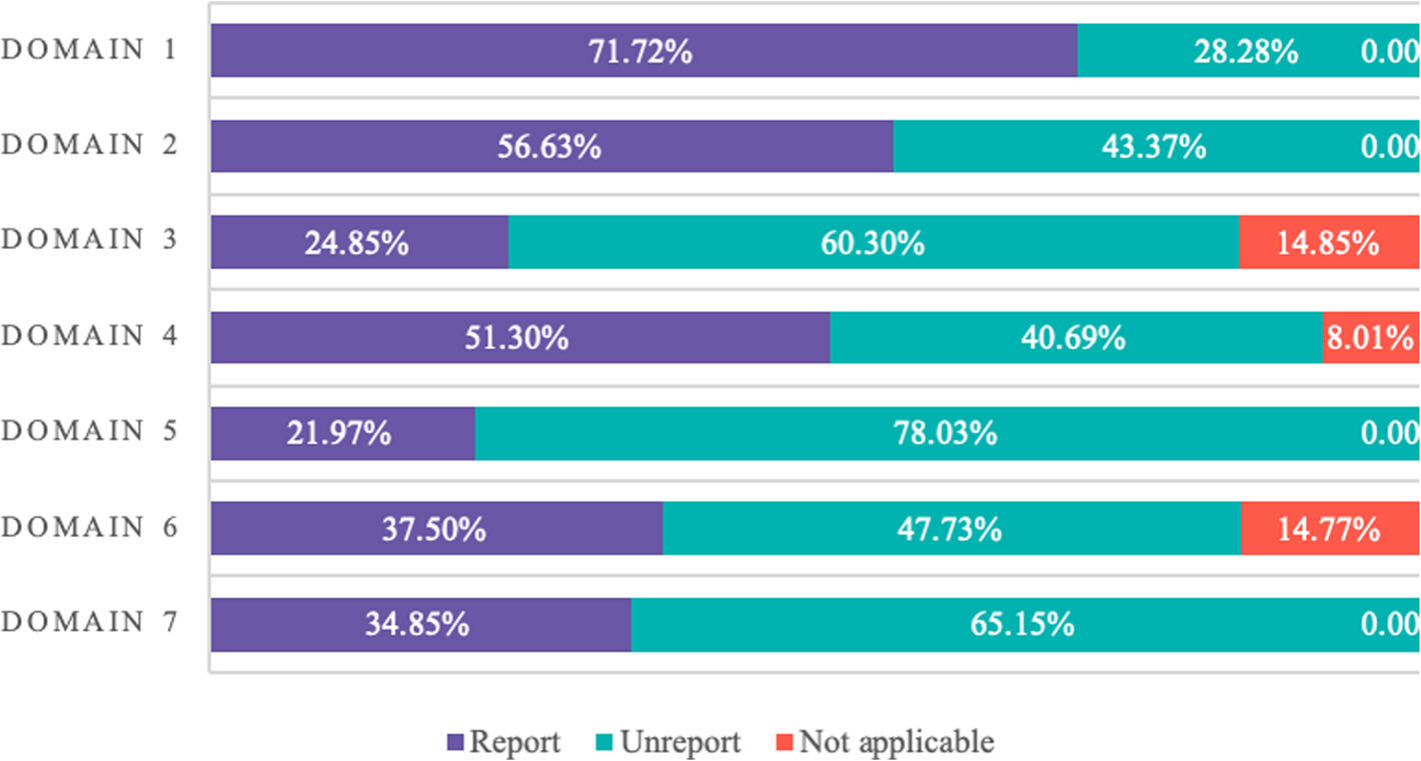
Graph representing the percentage distribution of scores for each domain in the RIGHT checklist for CPGs (*n* = 66)

Only 3 (4.6%) CPGs stated that they followed the guideline development tool, and all 3 followed the AGREE checklist. The 66 stroke CPGs were evaluated by 22 items in the RIGHT checklist with an average reporting rate of 47.6%. The reporting quality of each domain and each item are shown in Figs. 3 and 4 and Table 2.

**Table 2:** Distribution of scores and percentages for each item in the RIGHT checklist for the included CPGs (*n* = 66)

RIGHT checklist		Reported		Not reported		Not applicable	
		***N***	(%)	***N***	(%)	***N***	(%)
*Basic information*							
1a	Identify the report as a guideline, that is, with “guideline(s)” or “recommendation(s)” in the title.	64	97.0%	2	3.0%	0	0.0%
1b	Describe the year of publication of the guideline.	45	68.2%	21	31.8%	0	0.0%
1c	Describe the focus of the guideline, such as screening, diagnosis, treatment, management, prevention or others.	54	81.8%	12	18.2%	0	0.0%
2	Provide a summary of the recommendations contained in the guideline.	26	39.4%	40	60.6%	0	0.0%
3	Define new or key terms, and provide a list of abbreviations and acronyms if applicable.	42	63.6%	24	36.4%	0	0.0%
4	Identify at least one corresponding developer or author who can be contacted about the guideline.	53	80.3%	13	19.7%	0	0.0%
*Background*							
5	Describe the basic epidemiology of the problem, such as the prevalence/incidence, morbidity, mortality, and burden (including financial) resulting from the problem.	40	60.6%	26	39.4%	0	0.0%
6	Describe the aim(s) of the guideline and specific objectives, such as improvements in health indicators (e.g., mortality and disease prevalence), quality of life, or cost savings.	53	80.3%	13	19.7%	0	0.0%
7a	Describe the primary population(s) that is addressed by the recommendation(s) in the guideline.	32	48.5%	34	51.5%	0	0.0%
7b	Describe any subgroups that are given special consideration in the guideline.	10	15.2%	56	84.8%	0	0.0%
8a	Describe the intended primary users of the guideline (such as primary care providers, clinical specialists, public health practitioners, program managers, and policy-makers) and other potential users of the guideline.	42	63.6%	24	36.4%	0	0.0%
8b	Describe the setting(s) for which the guideline is intended, such as primary care, low- and middle-income countries, or in-patient facilities.	23	34.8%	43	65.2%	0	0.0%
9a	Describe how all contributors to the guideline development were selected and their roles and responsibilities (e.g., steering group, guideline panel, external reviewer, systematic review team, and methodologists).	48	72.7%	18	27.3%	0	0.0%
9b	List all individuals involved in developing the guideline, including their title, role(s) and institutional affiliation(s).	51	77.3%	15	22.7%	0	0.0%
*Evidence*							
10a	State the key questions that were the basis for the recommendations in PICO (population, intervention, comparator, and outcome) or other format as appropriate.	11	16.7%	55	83.3%	0	0.0%
10b	Indicate how the outcomes were selected and sorted.	5	7.6%	61	92.4%	0	0.0%
11a	Indicate whether the guideline is based on new systematic reviews done specifically for this guideline or whether existing systematic reviews were used.	22	33.3%	44	66.7%	0	0.0%
11b	If the guideline developers used existing systematic reviews, reference these and describe how those reviews were identified and assessed (provide the search strategies and the selection criteria, and describe how the risk of bias was evaluated) and whether they were updated.	12	18.2%	5	7.6%	49	74.2%
12	Describe the approach used to assess the certainty of the body of evidence.	32	48.5%	34	51.5%	0	0.0%
*Recommendations*							
13a	Provide clear, precise, and actionable recommendations.	38	57.6%	28	42.4%	0	0.0%
13b	Present separate recommendations for important subgroups if the evidence suggests that there are important differences in factors influencing recommendations, particularly the balance of benefits and harms across subgroups.	14	21.2%	21	31.8%	31	47.0%
13c	Indicate the strength of recommendations and the certainty of the supporting evidence.	55	83.3%	5	7.6%	6	9.1%
14a	Describe whether values and preferences of the target population(s) were considered in the formulation of each recommendation. If yes, describe the approaches and methods used to elicit or identify these values and preferences. If values and preferences were not considered, provide an explanation.	32	48.5%	34	51.5%	0	0.0%
14b	Describe whether cost and resource implications were considered in the formulation of recommendations. If yes, describe the specific approaches and methods used (such as cost-effectiveness analysis) and summarize the results. If resource issues were not considered, provide an explanation.	27	40.9%	39	59.1%	0	0.0%
14c	Describe other factors taken into consideration when formulating the recommendations, such as equity, feasibility and acceptability.	34	51.5%	32	48.5%	0	0.0%
15	Describe the processes and approaches used by the guideline development group to make decisions, particularly the formulation of recommendations (such as how consensus was defined and achieved and whether voting was used).	37	56.1%	29	43.9%	0	0.0%
*Review and quality assurance*							
16	Indicate whether the draft guideline underwent independent review and, if so, how this was executed and the comments considered and addressed.	26	39.4%	40	60.6%	0	0.0%
17	Indicate whether the guideline was subjected to a quality assurance process. If yes, describe the process.	3	4.5%	63	95.5%	0	0.0%
*Funding, declaration and management of interest*							
18a	Describe the specific sources of funding for all stages of guideline development.	27	40.9%	39	59.1%	0	0.0%
18b	Describe the role of the funder(s) in the different stages of guideline development and in the dissemination and implementation of the recommendations.	1	1.5%	26	39.4%	39	59.1%
19a	Describe what types of conflicts (financial and non-financial) were relevant to guideline development.	49	74.2%	17	25.8%	0	0.0%
19b	Describe how conflicts of interest were evaluated and managed and how users of the guideline can access the declarations.	22	33.3%	44	66.7%	0	0.0%
*Other information*							
20	Describe where the guideline, its appendices, and other related documents can be accessed.	27	40.9%	39	59.1%	0	0.0%
21	Describe the gaps in the evidence and/or provide suggestions for future research.	27	40.9%	39	59.1%	0	0.0%
22	Describe any limitations in the guideline development process (such as the development groups were not multidisciplinary or patients’ values and preferences were not sought), and indicate how these limitations might have affected the validity of the recommendations.	15	22.7%	51	77.3%	0	0.0%

Regarding the quality of reporting, the highest and lowest reporting rates were domain 1 (*basic information*, 71.6%) and domain 5 (*review and quality assurance*, 21.8%), respectively (Fig. 3). In addition, it is important to note that the overall quality of the report is affected by the fact that some of the items are not applicable in domains 3, 4, and 6 (Fig. 4).

**Fig. 5:**
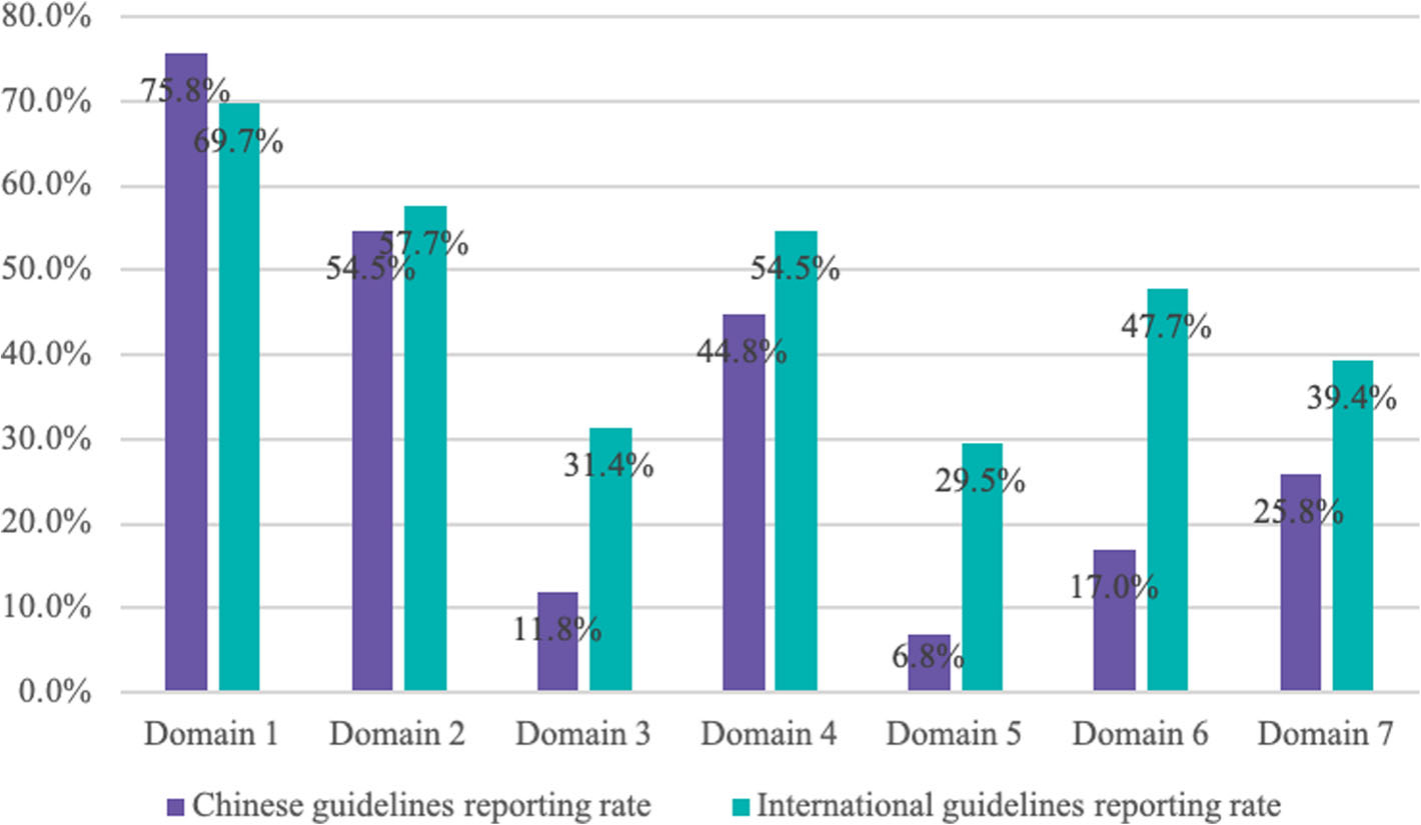
Comparison of adherence of RIGHT domains of Chinese and international stroke CPGs

In 7 domains of the RIGHT checklist, the largest difference in reporting rates between Chinese and International CPGs for stroke was 30.7% in domain 6 (funding, declaration, and management of interest) followed by domain 5 (difference of 22.7%) and domain 3 (difference of 19.6%). The smallest differences between Chinese and international CPGs were found in domain 1 (difference of 6.1%) and domain 2 (difference of 3.2%) with a higher reporting rate for Chinese than international CPGs in domain 1 (Fig. 5).

Of all the CPGs, 12 (18.2%) scored greater than 60%, and 8 (12.1%) scored less than 30%. Regarding each item, the results showed that items 1a (97.0%), 13c (83.3%), and 1c (81.8%) scored higher than the other items, whereas items 10b (7.6%), 17 (4.5%), and 18b (1.5%) were mainly distributed in the very low levels (Table 2).

### Subgroup analysis

**Fig. 6:**
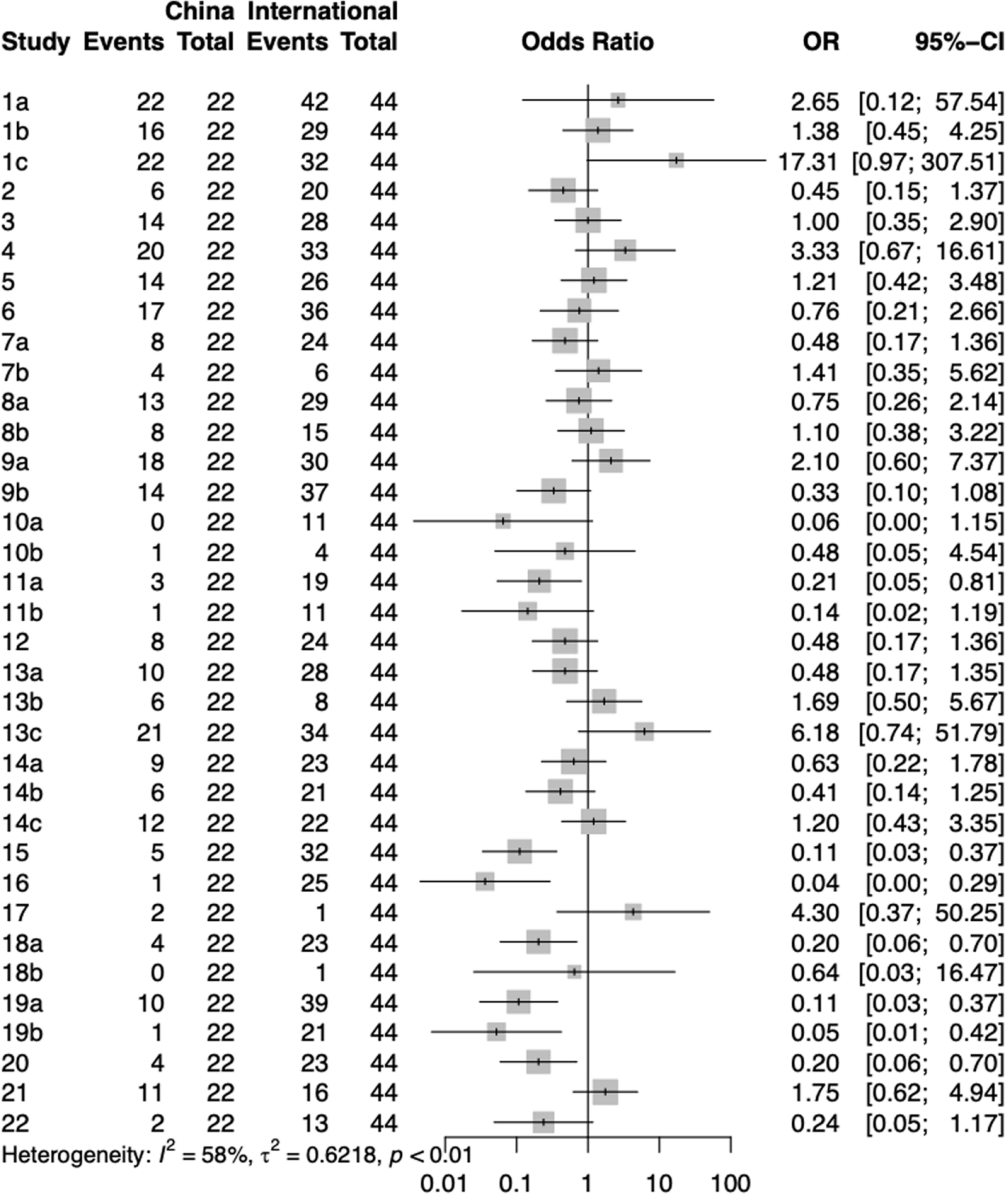
Comparison of different items between China and international CPGs

**Fig. 7:**
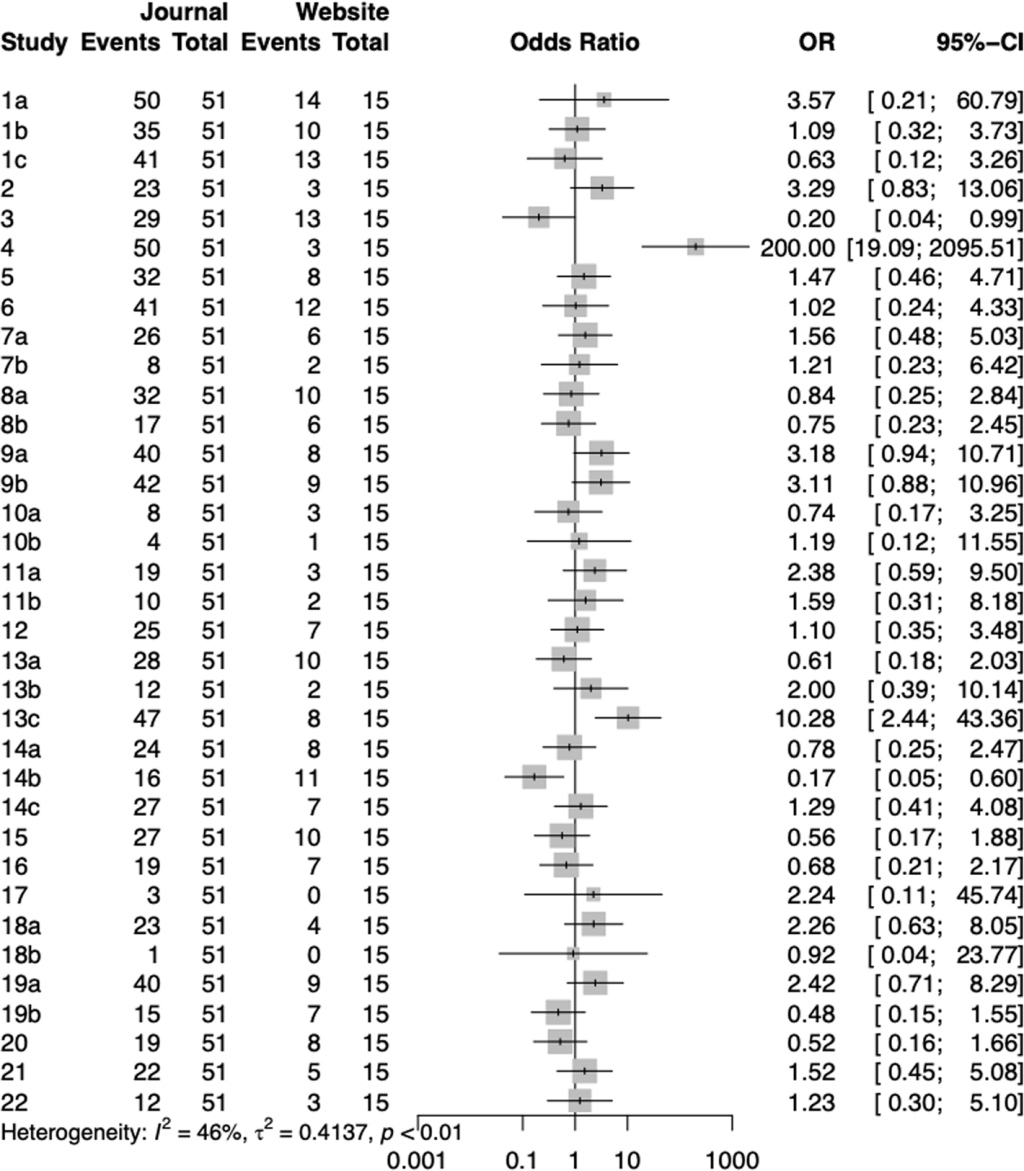
Comparison of different items between journal and website CPGs

**Fig. 8:**
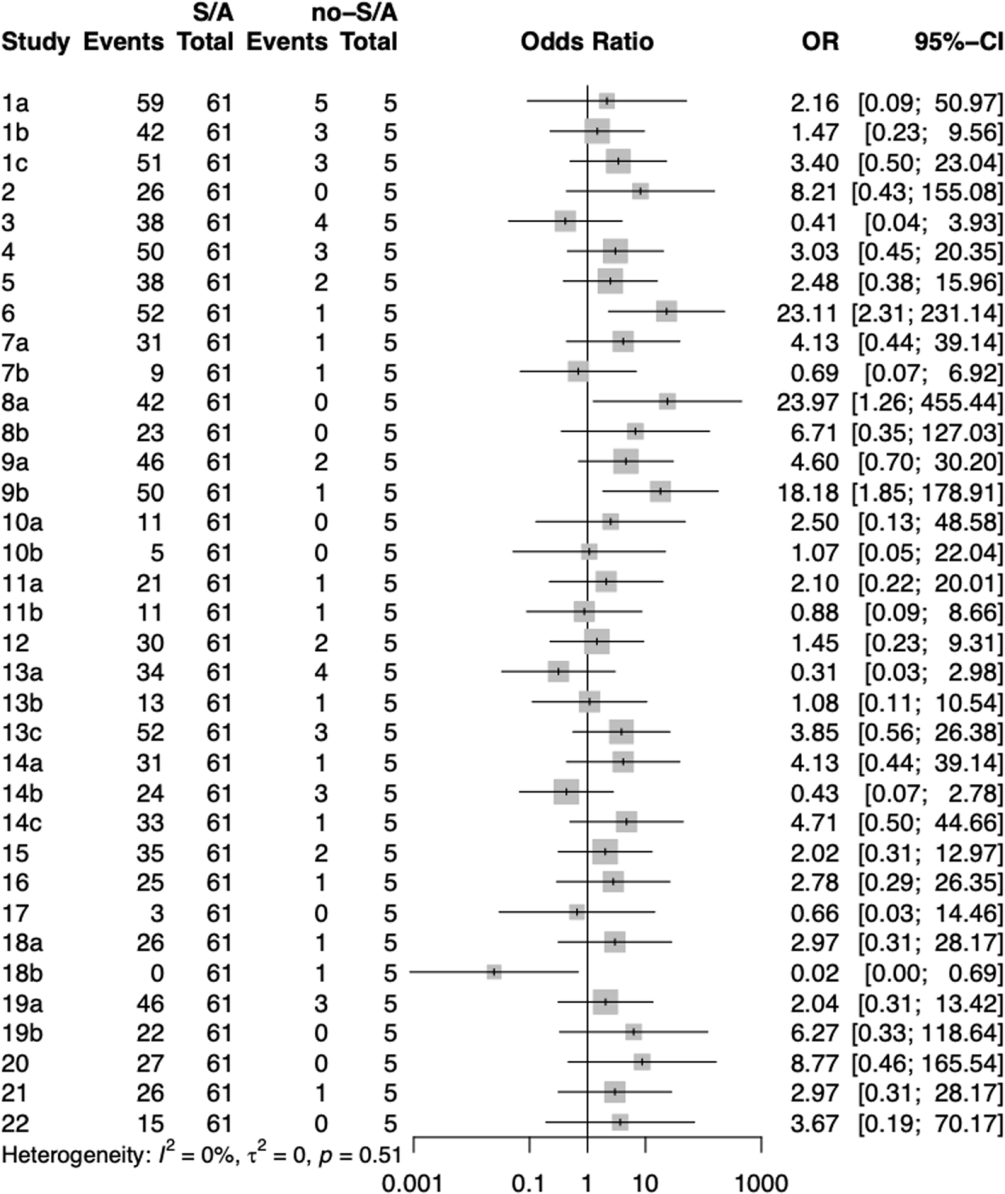
Comparison of different items between CPGs of societies/associations versus non-societies/associations

**Fig. 9:**
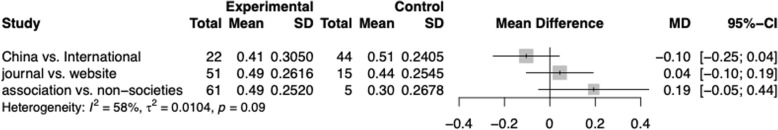
Forest chart for different subgroup CPGs for reporting quality

By comparing the reports of stroke CPGs under different classification methods, the following information was obtained (Figs. 6, 7, 8, 9):

The reporting quality of 22 CPGs published in China in the RIGHT checklist is lower than that of 44 published abroad, but the difference is not statistically significant. For items with statistically significant differences in reporting rates between China and International CPGs, the International CPGs have a higher reporting quality in items 11a, 15, 16, 18a, 19a, 19b, and 20.

The average reporting quality of the RIGHT checklist was similar in the CPGs published by journals compared with those published on the websites, and the difference was not statistically significant. In items 3 and 14b, CPGs published by journals were significantly lower than websites; however, in items 4 and 13c, the reporting quality of guideline published in journals was higher.

The average reporting quality of 61 CPGs developed by various societies OR associations was higher than that of 5 CPGs developed by non-societies OR associations without statistically significant differences. For specific items, the institute/association CPGs have a higher reporting quality in items 6, 8a, and 9b, whereas non-institute/association CPGs have the highest reporting quality in item 18b.

## Discussion

Our study is the first in the world to use the RIGHT checklist to evaluate the report quality of 66 stroke CPGs published in Chinese and international journals and networks over the past 5 years. Based on the 37-item RIGHT checklist, the average reporting rate of 66 CPGs was 47.6%. Domains 1, 2, and 4 had a higher reporting rates, whereas domains 3, 5, and 7 had the lower reporting rates. The significant preponderance of evidence-based CPGs indicates the overall high quality of stroke CPGs in the methodological field. This finding is consistent with the report quality evaluation results of CPGs for traditional Chinese medicine and CPGs for common public health diseases affecting the health of the Chinese population, such as diabetes [78, 79].

Two-thirds of the CPGs are ischemic stroke, and only a small proportion is specific for hemorrhagic stroke. This discrepancy may be due to the increased incidence and disease burden of ischemic stroke compared with hemorrhagic stroke. According to the *2019 China Statistical Yearbook of Health and Family Planning*, the incidence of ischemic stroke and disability-adjusted life expectancy increased yearly between 2005 and 2017, whereas the incidence of hemorrhagic stroke decreased yearly. In addition, the recurrence rate of ischemic stroke is higher, and the number of hospital discharges and per capita medical costs have dramatically increased.

We found here are much more Stroke CPGs in China compared to the rest of the world. This may be related to the aging of the Chinese population and the dramatic increase in stroke prevalence in rural China between 2003 and 2013. In conclusion, Chinese society is generally concerned about stroke.

Among stroke topics, treatment and management of CPGs are more common than prevention CPGs, which may be related to the emphasis on treatment over prevention in clinical practice. The INTERSTROKE study from 32 countries around the world showed that 90.7% of strokes worldwide were associated with 10 interventional risk factors [80]. For the Chinese population, these 10 risk factors were associated with 94.3% of strokes. This finding suggests that stroke is preventable and that primary prevention is a fundamental measure to reduce the incidence of stroke. However, current prevention guidelines of stroke focus more on secondary prevention. Stroke guidelines should pay more attention to prevention and management. In particular, there is a need to make efforts in the guidelines for the primary prevention of stroke.

Among the specific items, the reporting rates of 10b, 17, and 18b were very low, and only one guideline reported item 18b. Regarding item 18b, we found that the low reporting rate was not equal to low quality given the high rate of not applicable guidelines (54.9%). Taking the above into account, we decided to use the non-reporting rate as a proxy for the quality of the guideline. The unreported rates of 7b, 10a, 10b, and 17 items were all greater than 80%, reflecting the numerous shortcomings in the development of stroke CPGs in terms of consideration of subgroups, the key question statement in recommendations, selection of outcome indicators, and the assurance process of quality.

In item 7b, the CPGs were underreported at the subgroup level. However, the treatment and management were different in younger patients compared with older patients, and it is necessary to elaborate on age-specific measures by subgroup.

In item 10a, 83.3% of CPGs did not report key question in recommendations. According to the RIGHT checklist, development, such as PICO (population, intervention, comparator, and outcome), were recommended to state the key question statement in recommendations. The PICO format helps clinical and research professionals find the best available scientific information quickly and accurately when in doubt or question [81].

In item 10b, the outcome indicator is the carrier of evidence classification in the guideline. If the selection method of outcome indicator is not appropriate, it may lead to non-main outcomes, thus reducing the operability of the guideline.

Financial support is essential for the formulation of the CPGs, but it may be related to the conflict of interest in the formulation of the CPGs. The statements of financial support were unclear; guideline developers should document in detail the role of funding in the development, dissemination, and implementation of the CPGs to improve their feasibility and reliability.

According to the study [82], two CPGs published by the American Heart Association and the American Academy of Stroke in 2013 and 2018 involved a total of 34 authors, of whom 12 (35%) had financial conflicts of interest. Conflicts of interest are inevitable in the formulation of CPGs, so how to manage conflicts of interest is particularly important. The transparency of financial support is an important aspect. Therefore, it is recommended that experts improve the completeness of the reports of each item in the CPGs for stroke to improve the reliability and credibility of the CPGs for stroke clinical practice.

Based on the subgroup analysis of domestic and international CPGs, it cannot be concluded that the Chinese CPGs are significantly different from the international CPGs. Variances may be due to guide-setting practices, author habits, and health service models in China and abroad. In recent years, the development of evidence-based CPGs in China has followed the pace of international countries, and the literature and methodology of CPGs have also matured. However, there is a considerable gap with international guidelines in domain 6 (*funding, declaration, and management of interest*). Studies have shown that the quality of evidence-based ischemic stroke CPGs in China is greater than that of consensus-based Chinese CPGs and evidence-based and consensus-based Chinese CPGs [83]; China’s guideline development agency could enhance China’s capacity to provide or coordinate the synthesis of evidence for guideline development and monitor the work of guideline developers. China can use what is known and work with the international community to develop methodologies to address the challenges of evidence-based guidance development [84].

Comparing publication forms, it was found that the reporting quality of CPGs published in journals was similar to that of CPGs published on the website. The reporting rates of items 4 and 13c were much lower than those of the CPGs published in the journal. This finding may be attributed to the fact that the reports published on the website were less standardized and difficult to trace back to the source.

In addition, items 11b, 13b, and 18b in the checklist were excluded from the subgroup analyses due to their high discomfort rates. We do not consider these factors sufficient to affect the quality of our study.

In domain 2 (*background*), some societies or associations publish CPGs of higher quality than those of non-societies or associations. This finding may be due to a combination of more cutting-edge methodology, better staffing, and better institutional processes. The association or society has a better division of the labor system, more experience in developing CPGs, and therefore more strict control over quality. However, regardless of whether the CPGs were issued by an academy or association, the formulation of the CPG working group should be composed of multidisciplinary experts jointly involved in decision-making, in which the CPG methodological experts play a key role [85]. Excellent teamwork ensures the completeness of the report and mass stability.

## Conclusion

In summary, the quality of the existing CPGs for stroke reporting needs to be improved. Guideline developers need to follow relevant guideline reporting and evaluation tools (such as RIGHT), pay attention to the details in the process of guideline reporting and the comprehensiveness of the report, and strengthen the standardization of the guideline to better improve the quality of the guideline such that the guideline improves clinical practice.

## Data Availability

The datasets used and/or analyzed during the current study are available from the corresponding author on reasonable request.

## Abbreviations

RIGHT: Reporting Items for Practice Guidelines in Healthcare
PRISMA: Preferred Reporting Items for Systematic Reviews and Meta-Analyses
CPGs: Clinical Practice Guidelines
CVD: Cerebrovascular Disease
SDI: Socio-demographic index
GIN: Guidelines International Network
WHO: World Health Organization
NICE: National Institute for Health and Care Excellence
WSO: World Stroke Organization
